# Study on Stability of Mechanical Properties for Porous Fe-Cr-Al Alloys after Long-Term Aging

**DOI:** 10.3390/ma15103718

**Published:** 2022-05-22

**Authors:** Huibin Zhang, Junliang Ma, Zhencheng Gao, Fei Guo, Shenghang Xu, Guangya Hou, Guoqu Zheng

**Affiliations:** College of Materials Science and Engineering, Zhejiang University of Technology, Hangzhou 310014, China; 2111925011@zjut.edu.cn (J.M.); 2112025172@zjut.edu.cn (Z.G.); 2112025013@zjut.edu.cn (F.G.); shenghangxu@zjut.edu.cn (S.X.); houguangya@zjut.edu.cn (G.H.); zhenggq@zjut.edu.cn (G.Z.)

**Keywords:** FeCrAl, porous material, aging treatment, grain-boundary engineering, oxidation

## Abstract

Nowadays, both the ferrite phase and B2-structured intermetallic in the Fe-Cr-Al alloy system are developed as porous materials, which have been further applied as high-temperature filter materials in industry. This work presents a comparative study of the mechanical properties of porous Fe20Cr5Al, Fe10Cr10Al and Fe10Cr20Al aged at 480 °C for 500 h. The changes in tensile strength, elongation and hardness were determined, and the microstructure changes as well as slight oxidation states of the aged samples were investigated. The results show that the precipitated Cr-rich phase in porous Fe20Cr5Al can increase the hardness and decrease the ductility, while intergranular oxidation can degrade the mechanical performance of the three porous Fe-Cr-Al materials. It is noted that porous Fe10Cr20Al exhibits relatively superior mechanical stability during long-term aging. Meanwhile, by introducing boron, the mechanical performance of the aged porous Fe-Cr-Al alloys can be stabilized since the possible internal oxidation of the exposed grain boundaries is inhibited.

## 1. Introduction

Porous materials fabricated with Fe-Cr-Al series alloys have been widely developed into filter materials and catalyst supports applied in the fields of biomass gas purification [1], steam methane reforming [2], and solid oxide fuel cells [3,4] due to the merits of sound high-temperature mechanical properties, good manufacturability, and excellent heat and mass transfer capacity [5]. Porous Fe-Cr-Al alloys demonstrate excellent resistances to oxidation [6,7,8,9], sulfidation [9,10], carbonization, and molten salt corrosion [9,11] via forming a protective Al_2_O_3_ layer, thus showing promising prospects for replacing the porous ceramics. However, the long-term service of porous Fe-Cr-Al alloys at temperatures above 400 °C still suffers from the degradation of the mechanical property and structure stability. In view of this, the design of porous Fe-Cr-Al alloys with excellent stability is of far-reaching significance for promoting their applications.

Fe-Cr-Al series alloys actually include the FeCrAl alloys and the FeAl-based intermetallic compounds alloyed with Cr, which, respectively, correspond to the ferrite (α) phase region and intermetallic compound (α2) phase region of the Fe-Al-Cr ternary system [12,13]. The FeCrAl alloys are commercialized under the name FeCr alloy or Khantal [2]. Because the excessive addition of Al always deteriorates the mechanical properties [5,14] and hot workability of FeCrAl alloys [15,16,17,18], the metallurgy industry developed the FeCrAl alloy with content of Al of no more than 12%, and the content of Cr is up to 20%. The predicament of FeCrAl alloys is that their mechanical properties are vastly affected by the separation of the ferrite (α) phase. There are two equilibrium phases in the critical temperature range of 280–560 °C in the phase diagram of Fe-Cr, denoted as α and α′ phases, with Cr content ranging from 5 to 10 at.% Cr and 90–95 at.% Cr, respectively. Since the solid solution is thermodynamically unstable in the miscibility gap, when the thermal aging temperature of steel is in this region, the ferrite will undergo coherent separation and precipitate into the α phase and α′ phase, which induces the so-called 475 °C embrittlement [19,20,21]. Meanwhile, some studies [19,22,23,24] have shown that increasing the aluminum content and appropriately reducing the chromium content can significantly inhibit the precipitation of α′ phases in FeCrAl alloys during aging. Al addition seems to stabilize the α phase, probably due to the fact that Al can increase the interfacial energy in the early stage of phase separation and the formation energy in the later stage [23,24]. The FeAl-based intermetallic compounds are designed with relatively high content of Al and lower content of Cr. Although the FeAl intermetallic compound in polycrystalline form is brittle at room temperature, Cr alloying can improve its plasticity and toughness. Some studies [25,26] have pointed out that adding 5 wt.%~10 wt.% Cr to FeAl intermetallic compounds can improve the ductility and impact toughness. In addition, due to its lower Cr content and higher Al content relative to ferritic FeCrAl alloys, 475 °C embrittlement will not occur during intermediate-temperature aging.

Varying the content of Al and Cr not only influences the phase composition and mechanical properties of Fe-Cr-Al alloys, but also affects the structural stability by influencing the corrosion resistance [9,27]. It is known that the corrosion resistance of FeAl intermetallic material can be readily improved by increasing the Cr content to a certain extent [9,10]. Glasscock et al. [28] proposed that increasing the service life of porous FeCrAl could be achieved by enhancing the Al content since the formation of Al_2_O_3_ scales is at the expense of metal Al consumption. Besides the uniform corrosion, it is also noteworthy that the high-angle grain boundaries of porous metals are inherently vulnerable due to the highly connective and exposed porous structure. The grain boundaries can be the channels for the diffusion of O, thus resulting in the inner oxidation of grain boundaries [29,30]. To suppress such abnormal oxidation, grain-boundary engineering methods such as alloying with reactive elements (boron and RE metals) are usually employed [31,32]. In brief, the design of porous Fe-Cr-Al alloys with improved mechanical stability should consider their corrosion resistance at elevated temperatures seriously.

Up to now, both FeCrAl alloys and FeAl-based intermetallic compounds have been developed as porous materials for filtration applications [9,33,34,35]. This work aimed to design and conduct a comparative study for the high-temperature mechanical stabilities of porous ferrite FeCrAl and FeAlCr intermetallic when aged in an elevated-temperature environment. Meanwhile, boron (B) was introduced into the porous FeCrAl alloy to suppress the mechanical performance degradation caused by abnormal oxidation.

## 2. Materials and Methods

**Figure 1:**
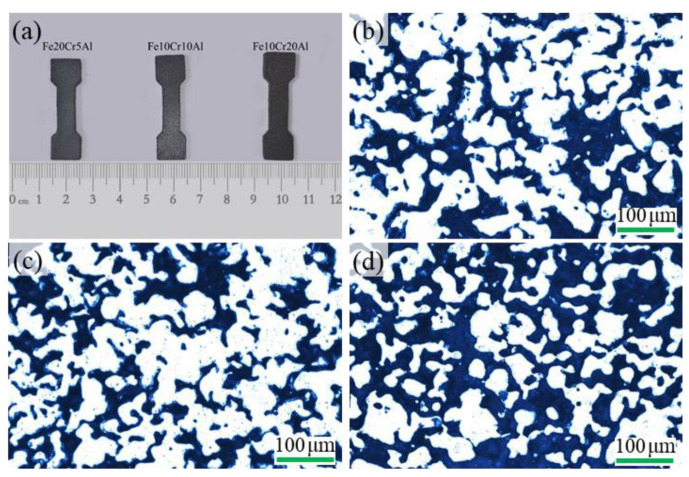
The bone-shaped tensile test samples (**a**) and the sectional view images of porous (**b**) Fe20Cr5Al, (**c**) Fe10Cr10Al and (**d**) Fe10Cr20Al.

The elemental powders of Fe (mean particle size ~50 μm, 99.5%), Al (~37.5 μm, 99.5%), Cr (~8 μm, 99.5%) and boron (particle size 1.0~3.0 μm, purity > 99.99%) were used as raw materials. The compositions of the three designed alloys are shown in Table 1. Fe20Cr5Al is a common FeCrAl alloy grade, while Fe10Cr20Al corresponds to the B2 structured FeAl intermetallic phase with Cr alloying [9]. The Fe10Cr10Al alloy with a moderate addition of Cr and Al was also assigned for a comparative study. In addition, 0.4 wt.% of boron was added based on the Fe20Cr5Al sample in order to improve the mechanical stability during aging. The raw powders were accurately weighed and mixed in a V-shaped mixer for 12 h. Pressure and sintering temperature affect the porosity and mechanical properties of porous materials. Low mold pressure and sintering temperature can reduce the mechanical properties of the sample, while an exceedingly high pressure and sintering temperature can lead to a sharp decline in the porosity of materials. In order to obtain materials with high porosity and well-developed sintering necks, we determined the appropriate pressure and sintering temperature based on previous experience [33]. The powder mixtures were pressed into bone-like compacts by mold pressing under a pressure of 200 MPa. The green compacts were then sintered in a vacuum furnace using the stepwise heating method to reduce the deformation of the samples caused by the violent reactions during heating [9], and the final sintering temperature was 1280 °C to obtain porous samples with uniform composition and structure. The samples were obtained after furnace cooling. The bone-shaped samples are shown in Figure 1a. The aging treatments were carried out in a tube furnace at 480 °C under a nitrogen (99.9%) gas flow of 1000 mL min^−1^. 

The sectional view images were observed using an optical microscope (Carl Zeiss, AG Axio Vert A1, Jena, Germany). Before optical microscopic observation, the samples were embedded into epoxy resin, followed by surface grinding with abrasive paper and polishing. The hardness was tested by a micro hardness tester (Shimadzu, HMV-G, Kyoto, Japan). Vickers hardness of polished samples was measured on the cross-sections of the porous skeleton. To exclude the influence of pores, indentations were made under a loading of 0.49 N. The phase composition was investigated using X-ray diffraction (Rigaku D/Max 2550 PC, Tokyo, Japan). Automatic step scanning was used to obtain the accumulation of a sufficient number of counts at (110) peak to obtain the desired statistical accuracy. At least 8 measurements were made on each sample. The microstructure of the samples was observed with a scanning electron microscope (Hitachi, Regulus 8100, Tokyo, Japan) equipped with energy-dispersive spectroscopy (Oxford, Ultim Max 65, Oxford, UK). Tensile tests were performed with a material testing system (INSTRON, 3369, Norwood, MA, USA) at room temperature. The porosity of the samples was determined using the Archimedes method. Transmission electron microscopy (FEI, TECNAI G2 F30 S-TWIN, Hillsboro, Oregon, USA) was employed to observe the evolution in alloy matrixes.

## 3. Results and Discussion

### 3.1. Characterization of the Synthesized Materials

Figure 1b–d display the sectional view images of the sintered porous Fe-Cr-Al materials with different compositions. In the optical images, the light regions are the porous skeleton and the dark regions are the pores. It can be seen that well-developed sintering necks and a connective porous structure have been obtained in the three porous alloys after sintering at 1280 °C. Compared with the interstitial pore-forming characteristic of the porous materials prepared with pre-alloyed powders, the pore structures of these materials possess unique features such as high connectivity and low tortuosity, which are inherited from the reactive synthesis method [9]. As shown in Table 2, the porosity of porous Fe-Cr-Al materials increases gradually with the increase in Al content. During reactive synthesis, due to the small difference in melting points between Fe and Cr, the partially asymmetric diffusion between them is not significant, while the melting point of Al differs markedly from those of Fe and Cr, so that partially asymmetric diffusion takes place vigorously between Al and Fe/Cr near the melting point of Al. Therefore, the interstitial pores among the original powders can be aggrandized by the partially asymmetric diffusion induced by Al-related reactions [33,35,36]. Correspondingly, the porosity of porous Fe-Cr-Al alloys can be improved by increasing the Al content. 

A more detailed description of the properties of the three materials is displayed in Table 2. It can be seen that with the increase in Al content, the tensile strength and fracture strain decrease and the hardness increases, suggesting the transformation from the ferrite (α) phase to the intermetallic phase.

**Figure 2:**
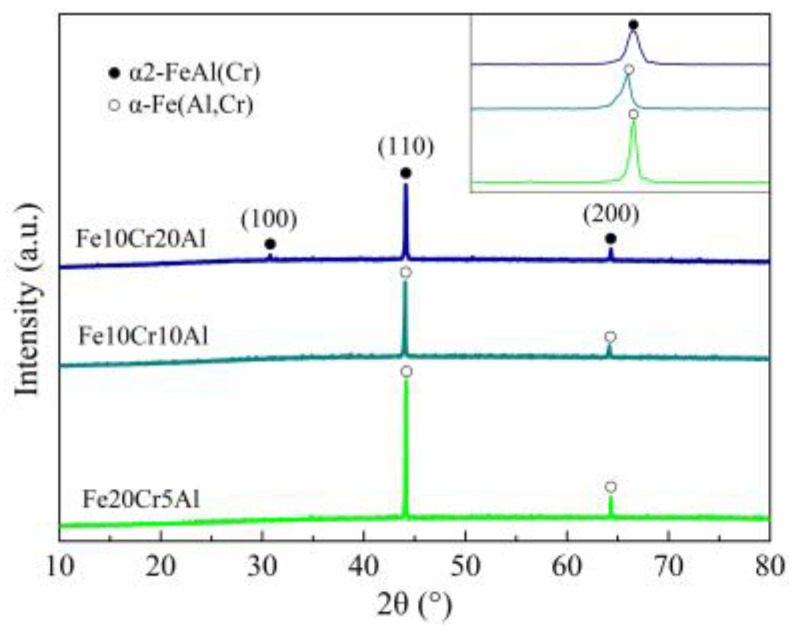
XRD patterns of the synthesized porous Fe-Cr-Al materials.

Figure 2 shows the XRD patterns of the synthesized porous Fe-Cr-Al materials. It is seen that the Fe20Cr5Al and Fe10Cr10Al samples can be indexed to the ferrite phase (α) with Cr and Al alloying, while the (100) peak of the Fe10Cr20Al sample appears at approximately 31°, implying that the increase in the Al content converts the α-ferrite phase into the ordered B2-structured FeAl phase (α2) with Cr alloying. Although both phases belong to a body-centered cubic structure, their lattice parameters vary with the content of Al and Cr. When the composition of Fe20Cr5Al changes into Fe10Cr10Al, the (110) peak shows a leftward shift since the atomic size of Cr is smaller than that of the Al element; meanwhile, the intermetallic phase has a more closely packed crystal structure, showing a decreased interplanar spacing. Moreover, we note an absence of any diffraction peaks of impurity phases, indicating that the alloying elements, such as Cr, completely enter in solid solution.

### 3.2. Evolution of the Material Properties during Aging

**Figure 3:**
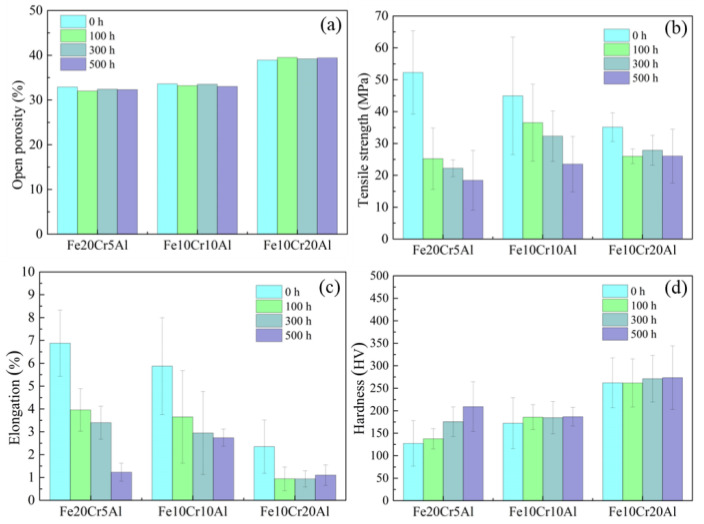
(**a**) Porosities, (**b**) tensile strength, (**c**) fracture elongation and (**d**) hardness of porous Fe-Cr-Al materials aged at 480 °C for different times.

The evolution of the open porosities of porous Fe20Cr5Al, Fe10Cr10Al and Fe10Cr20Al alloys was also tracked; see Figure 3a. It is seen that the porosities of these samples show only negligible changes after aging in N_2_ for 500 h at 480 °C, indicating that the growth of oxide scales is basically under control during aging.

Figure 3b,c depict the tensile testing results of porous Fe-Cr-Al materials after aging treatments for different times. In general, when increasing the content of Al and decreasing the content of Cr, the tensile strength and fracture strain of these synthesized porous alloys decrease. This can be attributable to the increase in porosity and the progress of the intrinsic brittleness of the intermetallic phase. With the increase in aging time, the strength and fracture strain of the three materials decrease significantly. Note that, after 500 h aging, the tensile strength of Fe20Cr5Al, Fe10Cr10Al and Fe10Cr20Al, respectively, decreases by 53.7%, 46.7% and 22.9%, and accordingly, the fracture strains, respectively, decrease by 81.8%, 53.4% and 52.1%. To be specific, the mechanical properties of the three materials decrease drastically in the first 100 h. However, in the next 400 h of aging, the tensile strength and fracture strain of Fe20Cr5Al and Fe10Cr10Al samples decline continuously, while those of Fe10Cr20Al samples remain almost invariable. Obviously, it can be found that porous Fe10Cr20Al corresponding to the single B2-structured intermetallic exhibits better mechanical stability during aging at intermediate temperatures.

Figure 3d shows the variations in the microhardness of the aged samples. It is seen that the hardness of the Fe20Cr5Al sample increases from 125 HV to 208 HV, while those of Fe10Cr10Al and Fe10Cr20Al only present slight changes after aging treatment. Such a difference also implies the occurrence of a discrepant change in the phase compositions of these different Fe-Cr-Al alloys. In ferrite FeCrAl alloys, the decomposition of primitive ferrite into the α and α′ phases usually takes place during aging, which can lead to the generation of stress and a compositional gradient, resulting in an increase in the hardness [12]. However, the variation in hardness is negligible in the intermetallic materials, suggesting that the Cr-rich phase is rarely generated during aging.

### 3.3. Mechanism for Mechanical Performance Degradation

#### 3.3.1. The Influence of Phase Separation

The relationship between the static strength and porosity of porous materials can be expressed by the following equation [36]:

(1) $$\sigma_{b}=\sigma_{0}{\left(1-\theta \right)}^{m}$$

$\sigma_{b}$ is the tensile strength of the porous material; $\sigma_{0}$ is the tensile strength of the corresponding dense material; m is a constant, depending on the material and manufacturing process; θ is the porosity of the testing material. As shown in Figure 3a, the porosities of these materials almost remain constant during the aging process, suggesting that the decrease in the apparent mechanical properties should be related to the degradation of the intrinsic properties of the alloys.

**Figure 4:**
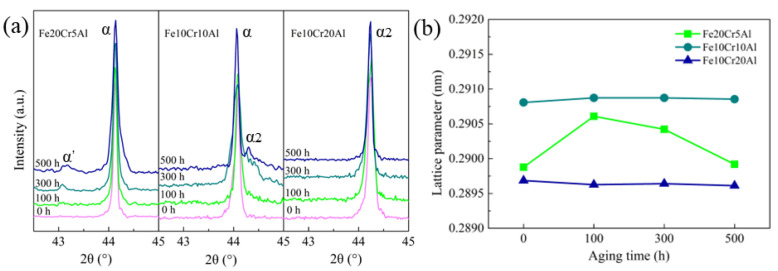
(**a**) The (110) diffraction profiles and (**b**) lattice parameters of porous Fe-Cr-Al materials after aging for different times.

In order to clarify the mechanism for the mechanical performance degradation of Fe-Cr-Al porous alloys, the microstructure evolution was investigated by step-scan XRD. Figure 4a shows the step-scan results, or more specifically, magnifications of the (110) diffraction profiles of the porous Fe-Cr-Al materials aged at 480 °C for 100, 300 and 500 h. For the Fe20Cr5Al samples after aging for 100 h, a wide sideband is observed at 43.1°and it becomes more intensive with the duration of aging. This seems to be attributed to the formation of the Cr-rich phase decomposed from the α phase [37]. Meanwhile, for the Fe10Cr10Al samples aged for 500 h, a sideband arises on the right side of the (110) peak. This sideband can be indexed to the B2 FeAl phase, indicating the occurrence of the ordering process. However, it is notable that the XRD patterns of the aged Fe10Cr20Al samples presumably remain in the initial state without sidebands, or the X-ray intensities of sidebands are very weak, suggesting that the crystal structure of Fe10Cr20Al intermetallic has higher stability upon aging at intermediate temperatures.

The lattice parameters of the three materials were determined using the software High-Score; see Figure 4b. It can be seen that the Fe20Cr5Al sample presents a slight change in lattice parameter. To be specific, the lattice parameters of Fe20Cr5Al samples aged for 0 h, 100 h, 300 h and 500 h are 0.2899 nm, 0.2906 nm, 0.2904 nm and 0.2899 nm, respectively, showing a trend of increase before decrease during the 500 h aging process. This can be caused by the precipitation of the Cr-rich phase (α′) since there is a diminutive deviation between the lattice parameters of the Cr-rich precipitates and the alloy matrix. At the beginning, the co-lattice nucleation of the Cr-rich phase can cause the matrix to generate a tensile stress and thus cause the increase in lattice. As the aging progresses, the Cr-rich phases form more uniform precipitates from the matrix, which reduces the tensile stress; thereby, the lattice parameter of the matrix decreases in reverse. However, as compared with the Fe20Cr5Al sample, the lattice parameters of the Fe10Cr10Al and Fe10Cr20Al samples almost remain constant, indicating that the microstructural changes in these two samples are imperceptible.

**Figure 5:**
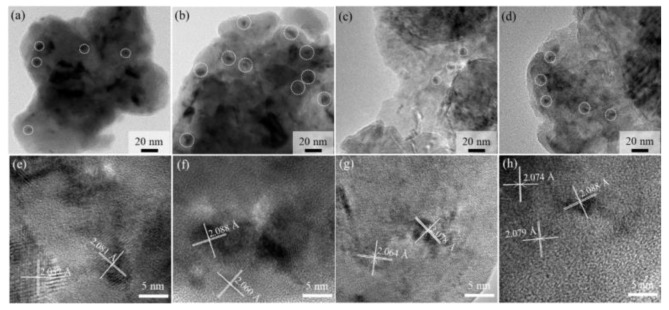
TEM images of porous Fe20Cr5Al after aging for 100 h (**a**,**e**) and 500 h (**b**,**f**) as well as Fe10Cr20Al after aging for 100 h (**c**,**g**) and 500 h (**d**,**h**).

TEM analyses were further conducted in order to reveal the microstructure evolution directly. Figure 5a,b show the TEM images of the porous Fe20Cr5Al alloy after being aged for 100 h and 500 h. Compared with Fe10Cr20Al samples, the Cr-rich phases (circled in Figure 5a–d) precipitated in the Fe20Cr5Al alloy after aging treatment are much more conspicuous. It is noteworthy that as the aging time increases from 100 h to 500 h, the precipitated α′ phases increase significantly in both quantity and size; some Cr-rich precipitates grow up and the lattice constant also becomes larger, which accords with the variation in the lattice parameter determined by XRD (Figure 5e,f). However, such a change is mild in the Fe10Cr20Al sample and seldom precipitated phases with an almost invariable lattice constant can be found; see Figure 5c,d,g,h. The result revealed by TEM verifies the XRD result, and also concurs with the previous works [12,23,38,39]. It is reported that the precipitation of α′ phases in bulk FeCrAl alloy (PM 2000) after aging at 475 °C caused a significant increase in both tensile strength and hardness with a concomitant decrease in elongation [39], which is merely opposite to the change in tensile strength in the present work. Therefore, the separation of the α phase in porous Fe20Cr5Al can only account for the decrease in ductility and the increase in hardness.

#### 3.3.2. The Influence of Oxidation

**Figure 6:**
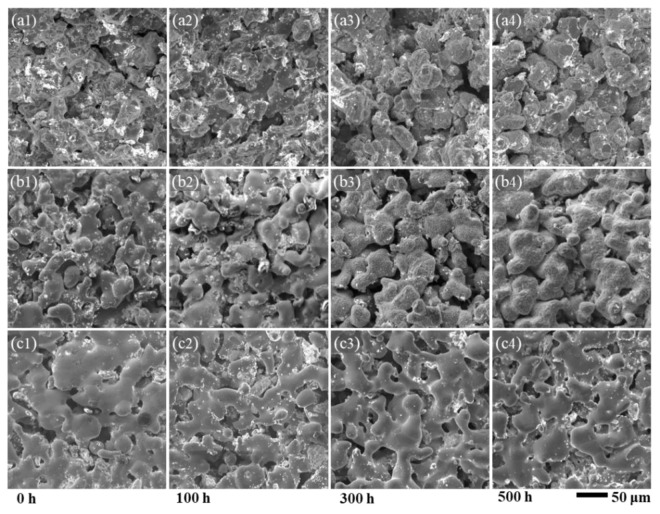
SEM images of porous Fe20Cr5Al (**a1**–**a4**), Fe10Cr10Al (**b1**–**b4**), Fe10Cr20Al (**c1**–**c4**) after aging for 0 h, 100 h, 300 h and 500 h, respectively.

**Figure 7:**
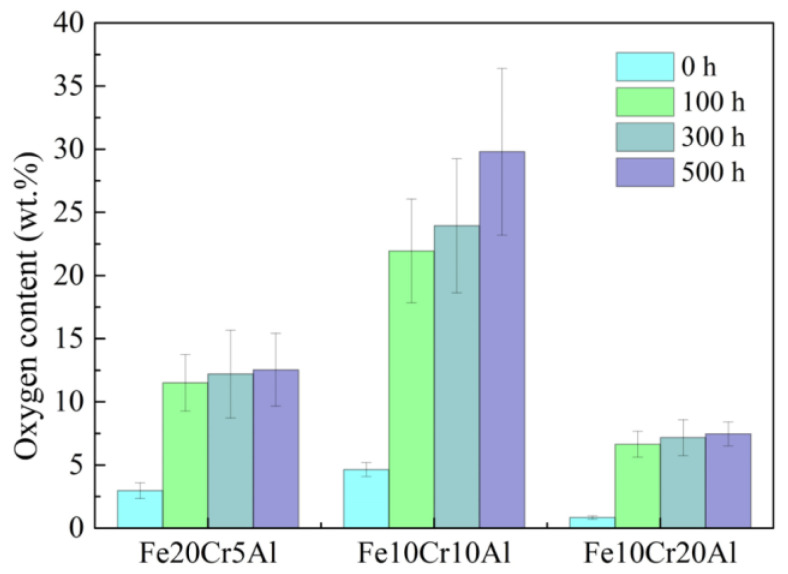
Oxygen content of the aged porous Fe-Cr-Al materials determined by EDS.

Although the aging process was carried out under a N_2_ atmosphere to avoid aggressive oxidation, slight oxidation actually took place on the testing samples due to the presence of residual oxygen that remained insides the pore structures or the trace leakage of oxygen into the heater tube. Therefore, we further studied the oxidation behaviors of these porous Fe-Cr-Al materials during aging. As shown in Figure 6a1–a4,b1–b4, during the 500 h aging process, obvious changes from smooth to rough porous skeleton surfaces were observed in Fe20Cr5Al and Fe10Cr10Al samples, indicating that the oxide scales were gradually formed and thickened. However, the oxides on the surface of the Fe10Cr20Al sample are almost invisible, and the grain boundaries can still be clearly observed in the sample aged for 500 h (Figure 6c1–c4). Through analyzing the surface oxygen content with EDS (Figure 7), it can be seen that the surface oxygen content of the three materials increased with the duration of aging time. In the first 100 h, there was a noteworthy increment in oxygen content. In the next 400 h aging process, the oxygen content of Fe20Cr5Al and Fe10Cr20Al samples only showed a limited increment, indicating that the generated oxide scales provided good protection, while the oxygen content of the Fe10Cr10Al sample continued to increase, indicating that the protection of the oxide film was relatively poor. Such discrepant oxidation performance is perhaps caused by the different ratios and compositions of Al and Cr in oxide scales [40]. To sum up, porous Fe10Cr20Al exhibits the superior resistance to oxidation.

**Figure 8:**
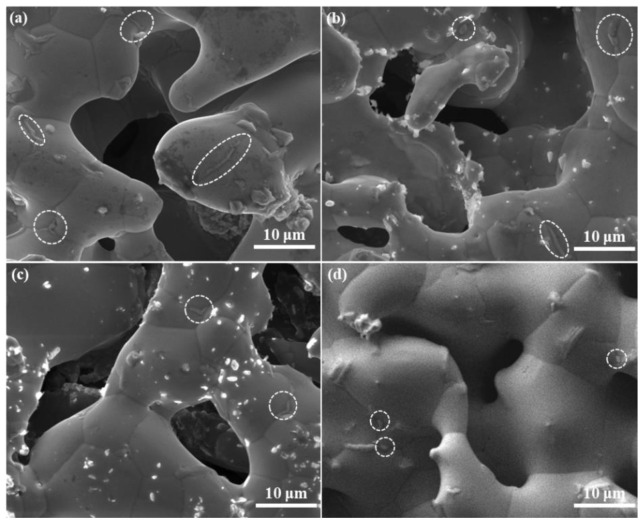
(**a**) SEM images of porous Fe20Cr5Al (**a**), Fe10Cr10Al (**b**), Fe10Cr20Al (**c**) and porous Fe20Cr5Al modified with 0.4 wt.% B (**d**) after aging for 100 h, respectively.

Comparing the changes in oxygen content and mechanical properties, it can be found that there is a certain correspondence between them, indicating that oxidation may be one of the reasons for the decrease in material strength. It can be seen from Figure 3b,c that the mechanical properties of these porous materials decrease significantly in the initial 100 h, which is closely related to the increase in oxygen content (Figure 7). However, the limited growth of oxide scales shown from the morphologies (Figure 6a2,b2,c2) of the 100 h aged samples indicates that the depletion of the metal porous skeletons is quite slight, which cannot result in a notable decrease in structure strength. Therefore, specific attention was given to the grain boundaries of porous Fe-Cr-Al samples after aging for 100 h. As shown in Figure 8a–c, some oxides (circled in Figure 8) nucleate at grain boundaries and grow into narrow strips or large particles along grain boundaries. This indicates that before the uniform oxidation of the surfaces, the highly exposed grain boundaries of the porous skeleton of Fe-Cr-Al materials have been attacked by the trace amounts of oxygen in the aging atmosphere. Accordingly, the weakened grain boundaries caused by intergranular oxidation may be the reason for the decrease in both tensile strength and elongations. Meanwhile, regarding the size of the oxides grown at grain boundaries in Fe20Cr5Al, Fe10Cr10Al and Fe10Cr20Al samples, it can be inferred that the intergranular oxidation of the Fe10Cr20Al sample is the slightest, which accords with its better mechanical stability in the later 400 h aging. Therefore, in terms of long-term high-temperature stability, porous Fe10Cr20Al shows the best service performance among the three studied materials. Moreover, Alumina fillers are needed to support the samples during sintering; thus, the shape of the samples can be maintained. Inevitably, some alumina debris was left and agglutinated to the surface of the samples. This will not significantly affect the performance of the samples.

Grain boundaries of metals are usually deemed as defects because of crystal misorientation and weak bonding [29,41]. Grain boundary oxidation will lead to a rapid decline in the strength and elongation of porous materials [42]. Therefore, grain boundaries can be rapid diffusion channels for both metal elements and oxygen at high temperatures. As for porous metals with highly exposed grain boundaries, the intergranular oxidation can lead to catastrophic damage to the mechanical performance. Accordingly, it is of significance for the protection of the grain boundaries of porous Fe-Cr-Al alloys. Some research [43,44] has revealed that some reactive elements, such as B or rare metals Ce and La, which segregate at grain boundaries, can be used to modify the grain boundaries by consuming the inward diffused oxygen and inhibiting the outward diffusion of metal ions. This work further introduced boron (B) to inhibit the oxygen erosion of grain boundaries.

**Figure 9:**
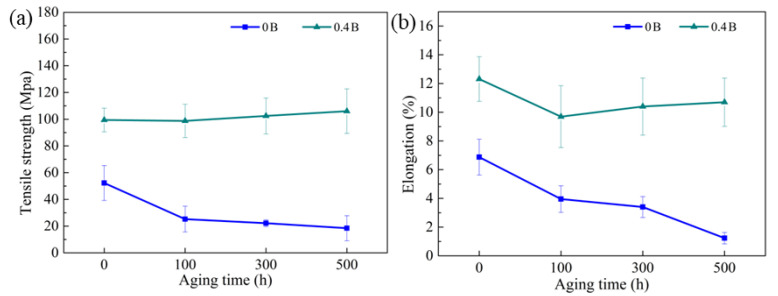
(**a**) Tensile strength and (**b**) elongation of porous Fe20Cr5Al alloy modified with 0.4 wt.% B after aging for different times.

Figure 8d shows the surface morphology of the 100 h aged porous Fe20Cr5Al with the addition of 0.4 wt.% B. Note that only a small amount of fine oxides grown at grain boundaries are found in this B-modified sample, indicating that the intergranular oxidation is restrained by B. As shown in Figure 9, the tensile strength and elongation of the B-modified porous Fe20Cr5Al sample are improved, which can be attributed to the solid solution and boundary segregation of B. It is suggested that introducing B can strengthen the substrate and grain boundaries via improving the density of valence electrons in the FeCrAl substrate and grain boundaries [43]. In particular, it can be seen that after the 500 h aging process, the mechanical properties of this material remain basically stable. To be specific, the tensile strength of porous Fe20Cr5Al even increases slightly, while the elongation decreases mildly, which accords with common aged bulk FeCrAl alloys excluding the influence of oxidation [39]. This result proves the conjecture that grain-boundary oxidation causes a decrease in the mechanical properties of porous Fe-Cr-Al alloys in this work.

**Figure 10:**
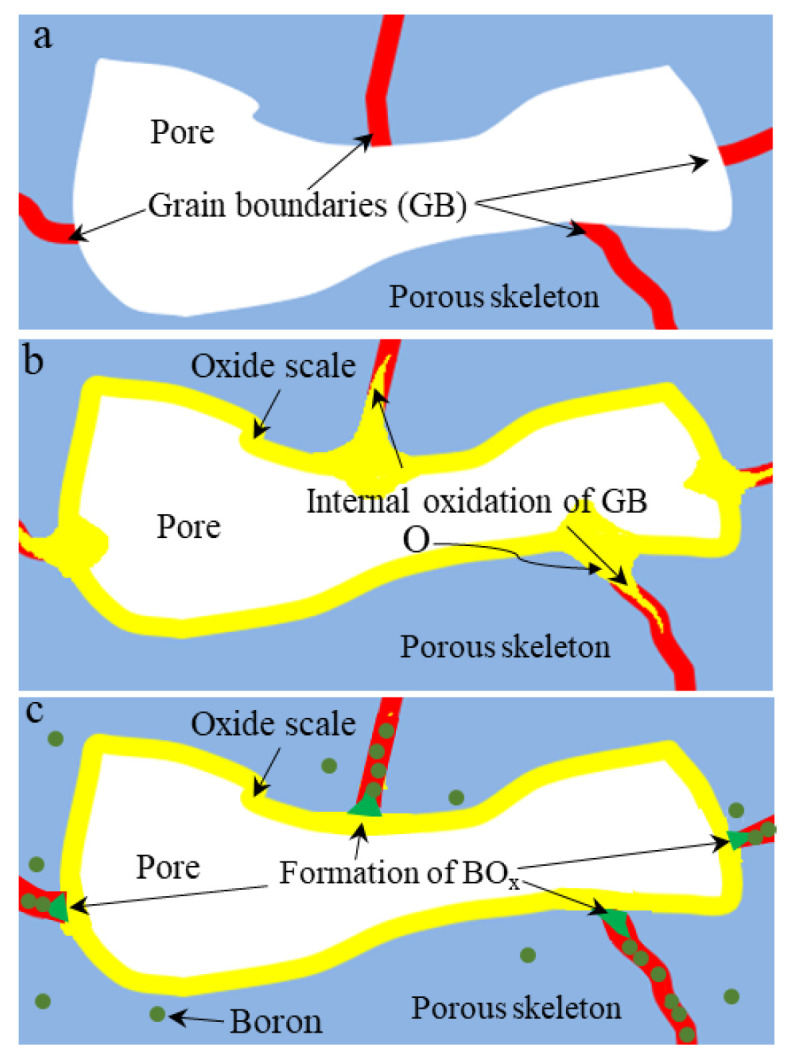
Schematic diagram for intergranular oxidation and the role of boron. (**a**) Porous material with highly exposed grain boundaries; (**b**) intergranular oxidation of porous materials; (**c**) formation of boron oxides inhibits inward diffusion of oxygen.

The intergranular oxidation of porous Fe-Cr-Al materials and the role of boron can be illustrated as in Figure 10. Because of the highly exposed grain boundaries in porous Fe-Cr-Al materials, the trace oxygen can attack the grain boundaries, causing the inward oxidation of the grain boundaries (Figure 10a,b). Thus, the mechanical properties, including tensile strength and elongations, are decreased during the aging process. B prefers to segregate in grain boundaries. It can not only strengthen the grain boundaries, but also consume the inward diffused oxygen through the grain boundaries, thus inhibiting the oxidation propagation of grain boundaries (Figure 10c) [32]. This work indicates that the introduction of B will also be a feasible way to improve the stability of the high-temperature mechanical properties of porous Fe-Cr-Al alloys.

## 4. Conclusions

Porous Fe20Cr5Al, Fe10Cr10Al and Fe10Cr20Al materials were prepared by reactive synthesis using mixed elemental powders, and their performance evolution during aging at 480 °C for a period of 500 h was investigated. During a period of 500 h aging, the tensile strength and elongation of porous Fe20Cr5Al and Fe10Cr10Al decrease continuously, while porous Fe10Cr20Al remains stable after the initial decline and shows the best mechanical stability against aging. The results show that the precipitated Cr-rich phase can lead to a decrease in elongation, and the intergranular oxidation is mainly responsible for the decline in tensile strength. By introducing boron, the possible abnormal internal oxidation of grain boundaries can be suppressed and thus the mechanical performance of porous FeCrAl alloys can be stabilized during aging.

## Figures and Tables

**Table 1:** Compositions of the studied Fe-Cr-Al alloys.

Element (wt.%)	Fe20Cr5Al	Fe10Cr10Al	Fe10Cr20Al
Fe	75	80	70
Cr	20	10	10
Al	5	10	20

**Table 2:** Properties of the synthesized porous materials.

Samples	Porosity%	Tensile Strength (MPa)	Strain%	Hardness (HV)
Fe20Cr5Al	33.1	52.3	6.9	127.3
Fe10Cr10Al	34.2	44.9	5.9	172.1
Fe10Cr20Al	39.4	35.1	2.3	261.8

## Data Availability

Not applicable.
